# *Serratia marcescens* Isolates from Bovine Mastitic Milk: Antimicrobial Resistance and Virulence Features

**DOI:** 10.3390/antibiotics14090892

**Published:** 2025-09-03

**Authors:** Guilherme Moreira, Luís Pinho, João R. Mesquita, Eliane Silva

**Affiliations:** 1 School of Medicine and Biomedical Sciences, ICBAS-UP, University of Porto, Rua de Jorge Viterbo Ferreira, nr. 228, 4050-313 Porto, Portugallapinho@icbas.up.pt (L.P.); jrmesquita@icbas.up.pt (J.R.M.); 2 Associate Laboratory for Animal and Veterinary Science (AL4AnimalS), 1300-477 Lisboa, Portugal; 3 Centro de Estudos de Ciência Animal (CECA), Instituto de Ciências, Tecnologias e Agroambiente (ICETA), University of Porto, Rua D. Manuel II, Apartado 55142, 4051-401 Porto, Portugal; 4 Research Center in Biodiversity and Genetic Resources (CIBIO/InBIO), University of Porto, Rua Padre Amando Quintas, nr. 7, 4485-661 Vairão, Portugal; 5 BIOPOLIS Program in Genomics, Biodiversity and Land Planning, CIBIO, Campus de Vairão, 4485-661 Vairão, Portugal

**Keywords:** bovine mastitis, *Serratia marcescens*, whole-genome sequencing, functional genes, phylogenomic analysis

## Abstract

**Background**: Bovine mastitis (BM) is a major disease affecting dairy herds (DHs), with *Serratia marcescens* (*S. marcescens*) being increasingly implicated as a causative agent. The growing concern over antimicrobial resistance (AMR) extends to BM-associated *S. marcescens* isolates, where resistance patterns are emerging. **Methods**: Here, four BM Gram-negative isolates were investigated: 1-DH1, 2-DH1, 3-DH2, and 4-DH3. Phenotypic characterization was performed using the Neg-Urine-Combo98 panel on a MicroScan WalkAway Plus system. Whole-genome sequencing (WGS) was performed to characterize and identify AMR and virulence factors (VF) genes and plasmids in isolates 1-DH1, 3-DH2, and 4-DH3, and phylogenomic analyses were conducted for a visual comparison of the genomes. **Results**: Phenotypically, isolates 1-DH1, 2-DH1, and 4-DH3 were identified as *S. marcescens*, and 3-DH2 as *Serratia odorifera* (confirmed as *S. marcescens* by WGS). A 28.00% (n = 25) prevalence of phenotypic AMR for isolates 1-DH1, 2-DH1, and 4-DH3 against Aug-E, AM, To, Cfx, Crm, Cl, and Fd was shown, and 24.00% (n = 25) for isolate 3-DH2 against Aug-E, AM, To, Crm, Cl, and Fd. The AMR genes *AAC(6′)-Ic*, *aac(6′)-Ic_1*, *aac(6′)-Ial*, *H-NS*, *SRT-2*, *oqxB*, *oqxB_1*, *oqxB25*, *mexI*, *CRP,* and *blaSST-1*, and *flgH*, *fliP*, *fliM,* and *fliG* VF genes were identified in the whole genome of the *S. marcescens* sequenced isolates 1-DH1, 2-DH1, and 4-DH3. In addition, a phylogenomic analysis of these three isolates revealed that WGS genomes are more closely related to *S. marcescens* prevenient from environmental sources. **Conclusions**: This study reports, for the first time, AMR resistance to tobramycin, cefuroxime, colistin, and nitrofurantoin in BM *S. marcescens* isolates. Genomic analysis revealed the presence of multiple AMR and VF genes, further highlighting the pathogenic potential of these isolates. Phylogenomic analysis revealed that the genome of the three BM *S. marcescens* isolates is more closely related to environmental *S. marcescens* strains.

## 1. Introduction

Bovine milk consumption is estimated at billions of liters worldwide, with the majority consumed as pasteurized milk [1]. However, the consumption of raw milk (RM) and unpasteurized dairy products poses a substantial public health risk, as they may be contaminated by pathogenic microorganisms originating from environmental sources or infected animals. Exposure to zoonotic pathogens through ingestion or direct contact can lead to zoonotic diseases (ZDs), with the consumption of RM widely recognized as one of the primary risk factors [1,2]. Nevertheless, non-zoonotic pathogens also pose a threat to animal health and welfare, and indirectly to public health, as they can serve as reservoirs of transferable antimicrobial resistance genes [3]. Overall, deliberately adopting the “One Health” concept within dairy herds (DHs) [4] could be a strategic framework for controlling ZDs, and good farming practices and effective post milk processing in a herd [1] could contribute to putting into practice both concepts.

Bovine mastitis (BM), defined as the inflammation of the mammary gland, is globally considered one of the most important diseases within DH due to its economic impact. It leads to considerable losses not only through decreased milk production and quality but also due to the reduced reproductive capacity of the affected animals, discarded milk, increased treatment costs, and higher culling rates [5,6,7]. Bovine mastitis can be classified as clinical (CM), subclinical (SCM), and chronic according to the degree of the mammary gland inflammation [8], and there are reported studies suggesting that SCM could account for more financial losses than CM in a herd [9,10]. Moreover, recent studies have described an increase in the incidence of environmental mastitis and a decrease in the incidence of contagious mastitis [11]. Regarding infections, various factors including pathogens, environmental factors, and opportunistic microorganisms are the most prevalent causes of BM in DH. The bacteria most commonly associated with BM include Gram-positive cocci such as *Staphylococcus aureus* (*S. aureus*), *Streptococcus agalactiae*, and *Streptococcus uberis*, as well as Gram-negative (GN) bacilli like *Escherichia coli* and *Klebsiella pneumoniae* [2]. Besides the mentioned pathogens, *Serratia marcescens* (*S. marcescens*) and *Proteus mirabilis* have been described as environmental causative agents of mastitis, and *S. marcescens* has been described as a causative agent of CM and SCM outbreaks [12,13]. Some cases of the SCM form may resolve spontaneously. However, there are reported studies where cows become chronic carriers of *S. marcescens* [14,15]. Additionally, *Serratia marcescens* is recognized as an emerging healthcare-associated pathogen, frequently implicated in nosocomial infections in humans. It primarily affects the respiratory and urinary tracts, as well as surgical wounds and soft tissues [16].

Regarding treatment, bovine mastitis (BM) remains a widespread infectious disease with a substantial economic impact, accounting for approximately 80.00% of antimicrobial usage in dairy animals. However, the global effectiveness of antimicrobial therapy has progressively declined, largely due to the emergence of novel bacterial resistance mechanisms. These mechanisms include the reduced permeability or antimicrobial uptake resulting from porin protein modifications; increased antimicrobial efflux mediated by efflux pumps; enzymatic inactivation of antimicrobials; alterations in antimicrobial targets via spontaneous point mutations; loss of enzymes responsible for antimicrobial activation; use of alternative metabolic pathways to bypass the need for inhibited targets; and acquisition of resistance determinants through mobile genetic elements [17,18,19], resulting in an increase in antimicrobial resistance (AMR) globally [3,16,20]. According to the literature, *Serratia marcescens* isolates have demonstrated resistance to multiple antimicrobial agents in BM cases, such as cefazolin, chloramphenicol, ampicillin, ceftriaxone, cefotaxime, and tetracyclin, and in human clinical infections, including resistance to penicillins, cephalosporins, tetracyclines, macrolides, nitrofurantoin, and colistin [21,22,23]. Moreover, several AMR genes detected in *S. marcescens* isolates from BM for extended-spectrum beta-lactamases (e.g., *bla*_TEM_, *bla*_CTX-M_, *bla*_CTX-M-15_, *bla*_CTX-M-14_, *bla*_CTX-M-65_, and *bla*_SHV-28_), chloramphenicol (e.g., *cmlA* and *floR*), carbapenem (e.g., *SIM-1*), and efflux pumps (e.g., *sdeB*, *sdeY*, *sdeR*, and *sdeD*) were previously described [21,23]. Furthermore, mutations in the *sdeS* efflux pump gene have been reported to upregulate the expression of *sdeAB* efflux pump genes, contributing to the development of multidrug resistance (MDR) in *S. marcescens* isolates, including those derived from aquatic environments [24]. In addition, the pathogenicity of *S. marcescens* is also mediated by an arsenal of virulence factors (VFs) including hemolysin, lipase, protease, prodigiosin, nuclease, chitinase, motility, and biofilm [25,26,27], and VF genes (e.g., *flhD*, *entB*, *kpn*, *mrkD*, *ycfM*, *bsmB*, *pigP*, *kfu*, and *shlB)* in *S. marcescens* isolates from BM have also been previously reported [21].

Antimicrobial resistance (AMR) is recognized as a major public health concern in Portugal, with several studies reporting prevalence rates ranging from 50.00% to 83.00%. These studies also describe the detection of AMR genes and multidrug-resistant (MDR) profiles, varying according to the study design and context. The reported resistant isolates include methicillin-resistant *Staphylococcus aureus* (MRSA), *S. aureus*, *Escherichia coli*, and *Enterococcus* spp., recovered from human, animal (including bovine mastitis), food-producing animal, and environmental sources [28,29,30,31,32]. In this context, our investigation focused on *Serratia marcescens* isolates obtained from cases of bovine mastitis.

## 2. Results

### 2.1. Phenotypic Identification and Antimicrobial Susceptibility Testing

Isolates 1-DH1, 2-DH1, and 4-DH3 were phenotypically identified as *Serratia marcescens* with a probability of 99.99%, while isolate 3-DH2 was identified as *Serratia odorifera* (*S. odorifera*) with a probability of 94.66%, using the Neg-Urine-Combo98 panel on the MicroScan WalkAway Plus system (Table 1). Regarding antimicrobial susceptibility testing (AST), *S. marcescens* isolates (1-DH1, 2-DH1, and 4-DH3) exhibited a phenotypic AMR prevalence of 28.00%, with resistance detected against Aug-E and AM (β-lactams), To (aminoglycoside), Cfx and Crm (cephalosporins), Cl (polymyxin), and Fd (nitrofuran). Isolate 3-DH2 showed a 24.00% phenotypic AMR prevalence, with resistance to Aug-E and AM (β-lactams), To (aminoglycoside), Crm (cephalosporin), Cl (polymyxin), and Fd (nitrofuran) (Table 1).

With respect to antimicrobial susceptibility, *S. marcescens* isolates 1-DH1, 2-DH1, and 4-DH3 demonstrated 72.00% phenotypic susceptibility to Cpe, Cft, Caz/CA, Etp, Imp, and Mer (β-lactams); NA, Cp, Lvx, and Nxn (quinolones); AK and Gm (aminoglycosides); T/S (sulphonamide); AZT (monobactam); Caz (cephalosporin); Fos (phosphonic acid derivative); Cft/CA (cephalosporin/β-lactamase inhibitor); and P/T (penicillin/β-lactamase inhibitor) (Table 1).

Similarly, isolate 3-DH2 exhibited a phenotypic AMR prevalence of 24.00% against the same antimicrobials, except for Cfx, to which it was not resistant (Table 1). Notably, 3-DH2 showed 76.00% susceptibility to the remaining tested antimicrobials (Table 1).

The minimum inhibitory concentration (MIC) values, interpreted according to EUCAST guidelines (as detailed in the Section 4), are presented in Table 1.

### 2.2. Genome Characteristics and Bacteria Classification

**Figure 1:**
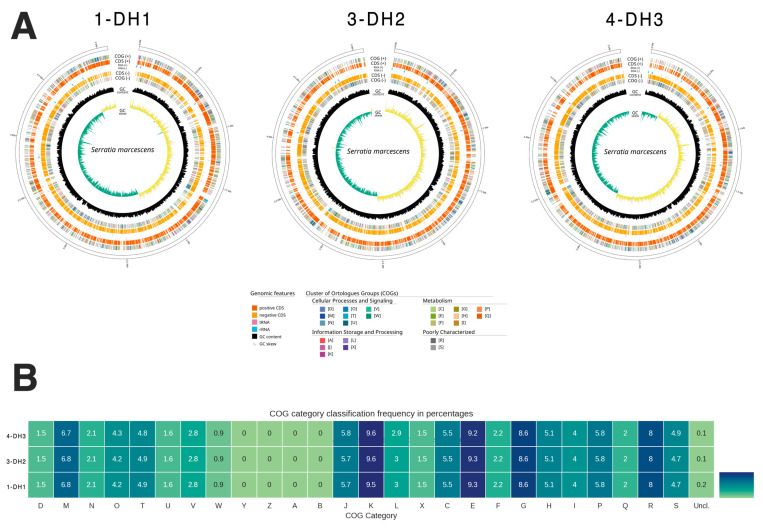
*Serratia marcescens* whole genome obtained for the three isolates, 1-DH1, 3-DH2, and 4-DH3, by WGS. (**A**) Whole-genome map of the three *S. marcescens* isolates, 1-DH1, 3-DH2, and 4-DH3. The median bacterial chromosome genome size was 5–5.1 Mb. The outer concentric circles (orange) indicate the coding sequence (CDS, +), the inner, dark yellow circle represents the CDS (-), the black circle indicates the % of GC content, and the inner circle indicates the GC skew [(G–C)/(G+C)], positive (light yellow) and negative (green). (**B**) Clusters of orthologous groups (COGs) of proteins category classification frequency in percentages obtained for the three isolates. The COGs obtained in the genome analysis of the three isolates by WGS could be seen in Table S1.

For the three whole-genome-sequenced bacteria isolates (isolates 1-DH1, 3-DH2, and 4-DH3), the median genome size was 5 Mb–5.1 Mb, with a G + C content of 59.50% and an average of 4727 protein-coding sequences. Mash [33] identified all the isolates as *S. marcescens*, with a 99.30% identity for all the three isolates (Table 2). The genome map and the clusters of orthologous groups (COGs) of the proteins obtained for the three isolates, 1-DH1, 3-DH2, and 4-DH, by WGS are shown in Figure 1.

### 2.3. Genomic Analysis of Antimicrobial Resistance Genes

The AMR genes *AAC(6′)-Ic*, *H-NS*, *tet(41)*, *SRT-2*, *oqxB*, *mexI*, and *CRP* were identified in whole-genome sequences of the *S. marcescens* isolates 1-DH1, 3-DH2, and 4-DH3, with identity percentages ranging from 97.10% to 100.00%, 80.85% to 100.00%, and 80.85% to 96.22%, respectively, by ABRicate using the CARD database (Table 3 and Table S2). Additionally, the AMR genes *aac(6′)-Ial*, *tet(41)*, *blaSST-1*, and *oqxB25* were identified in the genomes of all three isolates, with identity percentages ranging from 82.33% to 99.09%, as determined by ABRicate using the NCBI AMRFinderPlus database (Table 3 and Table S2). Furthermore, the genes *aac(6′)-Ic_1*, *tet(41)_1*, *blaSST-1_1*, and *oqxB_1* were identified in the three isolates with identity percentages ranging from 82.10% to 99.03% by ABRicate using the Resfinder database (Table 3 and Table S2). The antimicrobial resistance (AMR) genes identified were associated with specific resistance mechanisms and antimicrobial classes. The genes *AAC(6′)-Ic*, *aac(6′)-Ic_1*, and *aac(6′)-Ial*, which encode aminoglycoside-modifying enzymes, conferred resistance to aminoglycosides, specifically amikacin (AK) and tobramycin (To), through antimicrobial inactivation. The *H-NS* gene, involved in global gene regulation and functioning via an efflux-based resistance mechanism, was associated with resistance to cephalosporins, fluoroquinolones, macrolides, penicillins, tetracyclines, and cephamycins. The *tet(41)* gene, which also operates through antimicrobial efflux, conferred resistance to tetracyclines including acycline, doxycycline, and tetracycline. The *SRT-2* gene, encoding a β-lactamase, mediated resistance to cephalosporins, including ceftazidime (Cft), via antimicrobial inactivation.

Furthermore, the *oqxB* gene, associated with an efflux mechanism, was linked to resistance against diaminopyrimidines, fluoroquinolones, glycylcyclines, nitrofurans, and tetracyclines. Its variant, *oqxB_1*, conferred resistance specifically to nalidixic acid (NA) and ciprofloxacin (Cp). The *mexI* gene, part of the resistance–nodulation–division (RND) efflux system, was related to resistance to acridine dyes, fluoroquinolones, and tetracyclines. The *CRP* gene, a transcriptional regulator that modulates efflux pump expression, conferred resistance to fluoroquinolones, macrolides, and penicillins. Lastly, the *blaSST-1* gene, encoding a β-lactamase enzyme, conferred resistance to cephalosporins via antimicrobial inactivation. These findings are detailed in Table 3 and Table S2.

### 2.4. Genomic Analysis of Virulence Factors Genes

The virulence factor (VF) genes *flgH*, *fliP*, *fliM*, and *fliG*, which encode flagellar proteins, were identified in the whole-genome sequences of *Serratia marcescens* isolates 1-DH1, 3-DH2, and 4-DH3. These genes exhibited identity percentages ranging from 80.12% to 82.58% across all isolates, as determined using ABRicate (BLAST) against the VFDB database (Table 3 and Table S2).

### 2.5. Genomic Analysis of Plasmids

The *S. marcescens* whole-genome sequences of the three isolates (isolates 1-DH1, 3-DH2, and 4-DH3) were evaluated for the presence of plasmids using the PlasmidFinder tool and MOBScan server, and no plasmids were identified in any of the three isolates.

### 2.6. Phylogenomic Analysis

**Figure 2:**
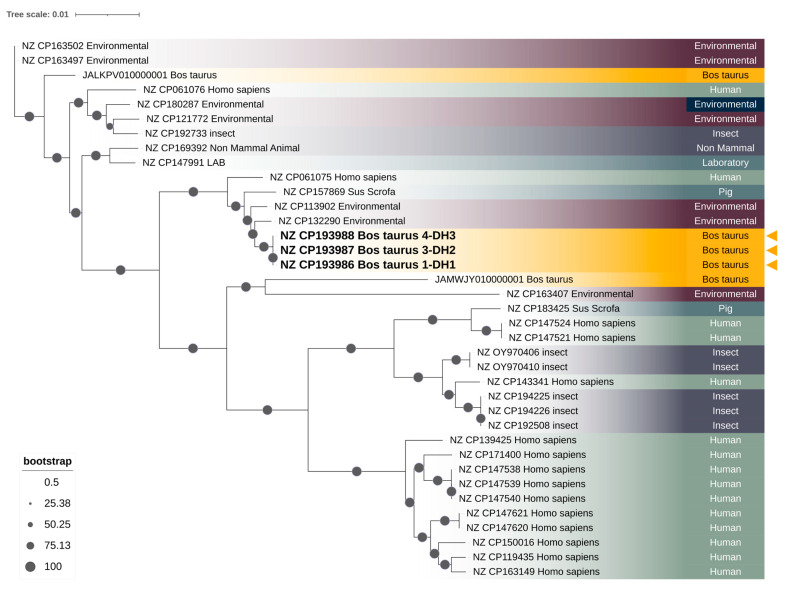
Phylogenomic analysis of the three *S. marcescens* whole-genome generated in this study, belonging to the *S. marcescens* isolates 1-DH1, 3-DH2, and 4-DH3 prevenient from BM. The phylogenetic tree was constructed based on pangenome core genes, as assigned by Panaroo, using the maximum-likelihood method based on the GTR + F + I + R8 model. *Serratia marcescens* sequences generated in this study are highlighted in bold.

A core genome phylogenetic analysis revealed two major and strongly supported *S. marcescens* clusters, each comprising isolates from multiple sources (Figure 2). The first cluster includes *S. marcescens* isolates of environmental, human, pig, and insect origin, as well as a single *Bos taurus* isolate (JALKPV010000001), and is supported by confident bootstrap values (100%). This cluster displays greater phylogenetic diversity overall, with longer internal branch lengths, reflecting a broader genomic divergence among its constituent isolates. Despite this diversity, isolates within the cluster exhibit relatively similar phylogenetic distances to each other when compared internally, suggesting a degree of lineage cohesion. However, this entire cluster is clearly separated from the rest of the tree by a longer basal branch, indicating a greater evolutionary distance from the second major cluster. This suggests that the isolates in the first cluster, while heterogeneous, share a more distant common ancestor relative to the isolates grouped in the second cluster.

In contrast, the second major cluster includes *S. marcescens* isolates from *Bos taurus* (including those sequenced in this study), as well as human, pig, and environmental origins. The three *Bos taurus* isolates (NZ_CP193986 *Bos taurus* 1-DH1, NZ_CP193987 *Bos taurus* 3-DH2, and NZ_CP193988 *Bos taurus* 4-DH3) generated in this study form a tightly grouped, strongly supported monophyletic clade (bootstrap: 99%), with near-zero branch lengths (0.00000003). This clade is embedded within a subcluster that also includes two environmental *S. marcescens* isolates (NZ_CP132290 and NZ_CP113902), forming a broader lineage with high bootstrap support (100%). The second cluster exhibits shorter branch lengths overall, particularly within the subcluster containing the study isolates, suggesting a lower genetic divergence compared to the first cluster. Additional subclusters within this second group include human *S. marcescens* isolates forming distinct branches with strong support, interspersed with pig and environmental genomes. 

The phylogenetic tree, thus, demonstrates the presence of genetically distinct lineages of *S. marcescens* with varying degrees of host and environmental association, and highlights the close genetic relationship among the *Bos taurus* isolates characterized in this study (Figure 2).

## 3. Discussion

Bovine mastitis is considered one of the most important diseases within DH worldwide due to its significant economic impact. This impact could be reflected in the decreased milk production and quality, reduced reproductive capacity, loss of discarded milk, and increased treatment costs and culling rates [5,6,7]. Recently, changes in the incidence of environmental and contagious mastitis have been reported, with increased cases of environmental mastitis and decreased cases of contagious mastitis [11]. The environmental pathogen *S. marcescens* has been described as a causative agent of CM and SCM outbreaks [12,13,14]. Furthermore, *S. marcescens* has been isolated from various farm environments, including bedding, milking parlors, feces of dairy cows, water, soil, different types of plants, and insects [34,35,36].

Bovine mastitis, as a widespread infectious disease, accounts for 80.00% of antimicrobial usage in dairy animals worldwide [37]. Moreover, the economic impact of mastitis and the risk of treatment failure highlights the significant financial burden on dairy farmers and the potential for the treatment to be ineffective [20,38].

Furthermore, AMR is also increasing due to new and emerging mechanisms of bacterial resistance that are spreading globally [3,20]. In this context, several studies have reported *S. marcescens* isolates from BM exhibiting AMR to antimicrobials such as cefazolin, chloramphenicol, ampicillin, ceftriaxone, cefotaxime, and tetracycline [21,23].

In this study, four GN bacterial isolates obtained from RM samples of BM cases from three DH in the northwest region of Portugal were investigated: isolate 1-DH1, isolate 2-DH1, isolate 3-DH2, and isolate 4-DH3. Using the Neg-Urine-Combo98 panel on the MicroScan WalkAway Plus system, isolates 1-DH1, 2-DH1, and 4-DH3 were phenotypically identified as *S. marcescens*, while isolate 3-DH2 was identified as *S. odorifera*. However, WGS confirmed isolate 3-DH2 as *S. marcescens,* indicating a misidentification by the panel-based method. The misidentification of isolate 3-DH2 highlights the inherent limitations of phenotypic methods in achieving precise species-level identification. Whole-genome sequencing (WGS), by providing comprehensive genetic information, enabled accurate taxonomic classification and unequivocally confirmed the isolate as *Serratia marcescens*. This genomic approach overcomes the ambiguities associated with phenotypic assays, thereby improving the reliability of bacterial identification in this study. To our knowledge, there are no published reports on the use of these panels for bacterial identification in RM from BM cases. Nonetheless, *S. marcescens* has been identified in human clinical samples using this technology with other panel types [39,40,41], suggesting that the panel used in this study or the others could be an alternative for *S. marcescens* identification and/or AST in RM samples from BM. Moreover, a study aimed at developing a direct method for rapid antimicrobial susceptibility testing and the identification of bacteria in positive blood cultures, including clinical and spiked isolates of *Serratia marcescens*, compared the performance of the matrix-assisted laser desorption ionization-time of flight mass spectrometry (MALDI-TOF MS), Vitek 2, and MicroScan WalkAway 96 Plus systems. The systems demonstrated categorical agreement rates of 94.90%, 97.40%, and 100.00% for MIC panels 53, 38, and MICroSTREP Plus 2, respectively. Comparable results were achieved across the tested platforms, as previously reported [41]. Regarding antimicrobial resistance (AMR) as determined by the Neg-Urine-Combo98 panel in the MicroScan WalkAway Plus system, *Serratia marcescens* isolates 1-DH1, 2-DH1, and 4-DH3 exhibited 28.00% phenotypic AMR prevalence to Aug-E and AM, To, Cfx, Crm, Cl, and Fd. These antimicrobials represent the β-lactam, aminoglycoside, cephalosporin, polymyxin, and nitrofuran classes, respectively. Based on these findings and in accordance with the literature, the isolates tested in this study can be classified as multidrug-resistant (MDR) bacteria [42]. Isolate 3-DH2, identified as *S. odorifera* by phenotypic testing but confirmed as *S. marcescens* via WGS, showed 24.00% resistance to the same set of antimicrobials, except for Cfx, showing additional susceptibility. Overall, the present findings suggest that antimicrobials such as Aug-E, AM, To, Cfx, Crm, Cl, and Fd may not be the most suitable options for treating BM caused by *S. marcescens.* Previously, the resistance of *S. marcescens* to Aug-E, AM, and Cfx had been described in BM, and the resistance to To, Crm, Cl, and Fd had been reported in human clinical isolates [21,22,43,44,45,46]. Resistance to To, Crm, Cl, and Fd were identified in this study. In terms of susceptibility, *S. marcescens* isolates 1-DH1, 2-DH1, and 4-DH3 (all 72.00%) and 3-DH2 (76.00%) were susceptible to the remaining tested antimicrobials. Overall, the antimicrobials Cpe, Cft, Caz/CA, Etp, Imp, Mer, NA, Cp, Lvx, Nxn, AK, Gm, T/S, AZT, Caz, Fos, Cft/CA, and P/T may represent better alternative treatment options for cows of all tested isolates (1-DH1, 2-DH1, 3-DH2, and 4-DH3). However, some of these agents are primarily intended for human use and are classified as critically important by the WHO or categorized as Category A by the EMA, which prohibits their use as veterinary medicines within the European Union. Nevertheless, antimicrobial susceptibility of *S. marcescens* to Cpe, Etp, Imp, NA, Cp, Lvx, Gm, T/S, Caz, Cft, Mer, AK, AZT, Fos, P/T, Cfx, and Cft/CA has previously been described in BM, as well as in human clinical outbreaks [21,22,44,45,47]. However, resistance to Cft/CA, Caz/CA, and Nxn has only been reported in human clinical cases [48,49,50]. On the other hand, emerging therapeutic alternatives for the control of BM, particularly, a wide range of natural products derived from plants, animals, and bacteria, have been reported to possess potential activity against BM and environmental causative agents of mastitis, including *Serratia marcescens*. These alternatives may provide added value in the management of BM and offer a strategy to circumvent the restrictions on the use of antimicrobials primarily intended for human medicine, supported by scientific evidence indicating their potential efficacy in treating BM [8,51,52,53,54]

Whole-genome sequencing (WGS) was performed for isolates 1-DH1, 3-DH2, and 4-DH3. Isolate 2-DH1 was not subjected to WGS as it originated from the same DH as isolate 1-DH1 and yielded comparable results in both bacterial identification and AST using the Neg-Urine-Combo98 panel on the MicroScan WalkAway Plus system. Based on the WGS analysis, all three sequenced isolates (1-DH1, 3-DH2, and 4-DH3) were identified as *S. marcescens*, with an average nucleotide identity of 99.30% compared to reference *S. marcescens* strains’ genomes. Moreover, the whole-genome analysis of the three *S. marcescens* isolates reveled hits for the AMR genes *AAC(6′)-Ic*, *aac(6′)-Ic_1,* and *aac(6′)-Ial* (all aminoglycoside acetyltransferase genes, conferring resistance to AK and To), *H-NS* (histone-like protein encoding gene *H-NS,* conferring resistance to cephalosporins, fluoroquinolones, macrolides, penicillins, tetracyclines, and cephamycins), *tet(41)* (tetracycline efflux pump gene, conferring resistance to tetracyclines), *SRT-2* (beta-lactamase gene, conferring resistance to Cft), *oqx, oqxB_1,* and *oqxB25* (RND efflux pump, conferring resistance to diaminopyrimidine, fluoroquinolone, glycylcycline, nitrofuran, tetracycline, phenicol, quinolone, NA, and CP), *mexI* (membrane transporter gene, conferring resistance to acridine_dye, fluoroquinolone, and tetracycline), *CRP* (multidrug efflux pump repressor gene, conferring resistance to fluoroquinolone, macrolide, and penicillin), and *blaSST-1* and *blaSST-1_1* (cephalosporin-hydrolyzing class C beta-lactamase gene, conferring resistance to cephalosporins) with identity percentages ranging from 80.85% to 100.00%. The AMR genes *AAC(6′)-Ic*, *aac(6′)-Ic_1*, *aac(6′)-Ial*, *H-NS*, *SRT-2*, *oqxB*, *oqxB_1*, *oqxB25*, *mexI*, *CRP*, and *blaSST-1* have been previously reported in *S. marcescens* isolates from clinical human and environmental sources [50,55,56,57,58,59,60,61]. However, to the best of our knowledge, this is the first report of these genes in *S. marcescens* isolates associated with BM outbreaks, suggesting a novel finding in the context of animal health and dairy production. The resistances potentially conferred by these genes correspond only to the phenotypic resistance observed for To and Fd in the four tested isolates 1-DH1, 2-DH1, 3-DH2, and 4-DH3. However, such discrepancies are unfortunately common, and a similar phenomenon has been previously reported in *Serratia sarumanii* [62]. Moreover, *S. marcescens* is also described as intrinsically resistant to Fd [63] and the presence of *oqxA/B* genes linked to Fd AMR were previously reported in clinical Enterobacteriaceae [64]. Relative to the *tet(41)* AMR gene, it was previously described in *S. marcescens* from clinical human and BM isolates, and also in environmental isolates [21,65]. The AST for this antimicrobial was not performed, as it is not included in the used panel (Neg-Urine-Combo98 panel) for GN bacteria identification and AST, although the presence of the gene suggests tetracycline resistance. Overall, the observed AMR determinants are chromosome-borne, as they were identified within the chromosomal sequences of the whole-genome assemblies of the three *Serratia marcescens* isolates studied. Furthermore, the discrepancy observed between phenotypic and genotypic resistance profiles may be attributed to factors such as gene expression regulation, variability in efflux pump activity, or gene silencing, consistent with previous reports [56,61,66,67].

Regarding the VF genes *flgH*, *fliP*, *fliM,* and *fliG* (also chromosome-borne) homologous to flagella proteins of *Yersinia enterocolitica* subsp. *Enterocolitica*, they were identified in the genome sequences of the *S. marcescens* isolates 1-DH1, 3-DH2, and 4-DH3, with identity percentage ranging from 80.12% to 82.58%. These VF genes were previously described in *S. marcescens* or *Serratia* species from clinical human isolates [50,68]. These are not reported in *S. marcescens* from clinical BM isolates in the literature, suggesting that they are reported for the first time in the present study. Moreover, these VF genes were previously reported in *S. marcescens* isolates linked to biofilm production [27], and, as flagella are involved in bacterial adhesion and invasion both indirectly by providing motility towards target cells and receptors, and directly by adhering to these targets, the possibility of mammary gland tissue invasion and adhesion for biofilm formation is high, contributing to ineffective antimicrobial treatment or resulting in persistent infections [69,70,71,72,73,74]. Additionally, plasmid screening in the three sequenced *S. marcescens* genomes revealed no plasmids, as no extrachromosomal DNA was detected. This suggests that our isolates may lack mobile genetic elements conferring antimicrobial resistance (AMR), in agreement with the previous findings reported in the literature [75].

Concerning the phylogenetic analysis, the core genome phylogeny revealed that the three *S. marcescens* isolates obtained from *Bos taurus* in this study (NZ_CP193986 *Bos taurus* 1-DH1, NZ_CP193987 *Bos taurus* 3-DH2, and NZ_CP193988 *Bos taurus* 4-DH3) form a tightly clustered, strongly supported monophyletic group (bootstrap: 99%), with near-zero branch lengths (0.00000003). This pattern indicates a clonal lineage with very recent divergence. The bovine-associated clade is nested within a broader lineage that includes environmental *S. marcescens* isolates (NZ_CP132290 and NZ_CP113902), separated by short genetic distances (≤0.003), which suggests a recent common ancestry and supports the hypothesis of an environmental origin for the isolates described in this study. The close phylogenomic relationship between these bovine and environmental *S. marcescens* isolates may reflect the capacity of certain environmental strains to colonize or infect mammalian hosts under suitable conditions [14,76,77]. Furthermore, the three *S. marcescens* isolates from this study are phylogenetically distinct from isolates originating from human, insect, non-mammalian, laboratory, and pig outbreaks, as well as from other environmental and *Bos taurus* isolates. Notably, they also diverge considerably from the previously reported *Bos taurus S. marcescens* isolate JALKPV010000001 (BM-2019), which clusters within a separate, distantly related lineage comprising primarily insect and human *S. marcescens* isolates. The phylogenetic separation of JALKPV010000001 suggests it may represent an independent acquisition from an unrelated lineage, highlighting the genomic diversity of *S. marcescens* strains associated with cattle. These results point to the presence of at least two genetically distinct *S. marcescens* lineages capable of colonizing *Bos taurus*: one more closely related to environmental and mammal isolates, as observed in the genomes sequenced in this study, and another more distantly related lineage with distinct ecological associations. Additionally, it is important to note that the core genome phylogeny focuses on genes conserved across all isolates, providing a stable and reliable framework for reconstructing evolutionary relationships, especially for identifying clonal lineages. However, this approach also has limitations. By excluding the accessory genome, the analysis may overlook critical genetic differences relevant to pathogenicity and ecological fitness. Accessory genes are frequently carried on mobile genetic elements, and their presence or absence can greatly influence the phenotypic diversity among isolates that appear closely related based solely on the core genome. Furthermore, horizontal gene transfer events, a major driver of bacterial evolution, are often missed in core genome analyses, potentially underestimating recent adaptive changes [78]. Supporting this hypothesis, several studies have demonstrated that environmental reservoirs on farms are frequent sources of *S. marcescens* associated with BM. Moreover, *S. marcescens* has been isolated from bedding pack materials, such as sawdust, which can serve as a reservoir for infection, and genetic analysis has shown that strains from bedding and infected milk are often identical, confirming bedding material as a direct source of BM outbreaks [36,79]. Contaminated post-milking teat dip solutions and temporary containers have also been identified as significant sources of *S. marcescens*. Contamination typically arises at the farm level—unopened commercial products usually test negative, while containers handled on-farm often test positive [79]. The milking machine itself can facilitate the transmission of *S. marcescens* from environmental sources like sawdust to the udder, especially when contaminated material is aspirated during milking [79]. In contrast, *S. marcescens* isolates derived from humans, which are predominantly associated with nosocomial infections, form multiple distinct and genetically diverse lineages, with longer branch lengths and no immediate clustering with the bovine strains. Insect-associated *S. marcescens* strains were phylogenetically variable, with some forming discrete clades and others interspersed among environmental or clinical isolates, implying a possible role as environmental vectors or reservoirs [80]. Overall, the phylogeny supports a scenario in which the mastitis-causing *S. marcescens* isolates may have evolved from environmental ancestors, distinct from human-associated hospital strains, with a possible host adaptation to cattle [76,81].

On the other hand, exposure to zoonotic pathogens through ingestion or direct contact can lead to zoonotic diseases (ZDs), with the consumption of RM being widely recognized as one of the primary risk factors [1,2]. Within the framework of the One Health concept, several studies have investigated the potential transmission of *S. marcescens* zoonosis in various contexts, including the environment, food, animals (including bovine milk, and BM), materials (including DH), and humans contaminated or infected with this pathogen. This highlights the need for continued research on *S. marcescens* in these areas to prevent its dissemination and support the control of BM, contributing to animal and public health [14,82,83,84].

## 4. Materials and Methods

### 4.1. Samples

Four GN bacterial isolates derived from RM samples of four cows with BM from three DH in the Entre-Douro e Minho region of northwest Portugal in February 2025 were used in this study. These isolates are part of the microorganism collection of SVA Expleite, Ldª, Fradelos, Portugal. Isolates 1 and 2 belong to DH1 (1-DH1, 2-DH1), isolate 3 to DH2 (3-DH2), and isolate 4 to DH3 (4-DH3). Isolate 1-DH1 is from a cow with SCM (with 1874 somatic cell count), isolates 3-DH2 and 4-DH3 are from cows with CM (both with >5000 somatic cell count), and it was not possible to further characterize the BM associated with the isolate 2-DH1.

### 4.2. Isolate Identification and Antimicrobial Susceptibility Testing

The four GN bacterial isolates were streaked onto Agar MacConkey (Merck, Darmstadt, Germany) and incubated at 37 °C for 24 h. Following incubation, bacterial identification and antimicrobial minimum inhibitory concentration (MIC) testing of the grown colonies were performed using the Neg-Urine-Combo98 panel (both tests in 1 run) (Beckman Coulter, Tokyo, Japan) using a MicroScan WalkAway Plus following the MicroScan Gram-negative procedural manual instructions (Beckman Coulter, Tokyo, Japan), as previously described [47], including European Committee on Antimicrobial Susceptibility Testing (EUCAST) and Clinical and Laboratory Standards Institute (CLSI) guidelines for AST interpretation [85,86,87]

The antimicrobials included in the panels and the MIC dilutions for antimicrobial interpretation, respectively, are as follows: amikacin (AK, 8-16), amoxicillin-clavulanate acid (Aug-E, 8/2, 32/2), ampicillin (AM, 4-8), aztreonam (AZT, 1-4, 16), cefepime (Cpe, 1, 4-8), cefotaxime (Cft, 1-16), cefotaxime-clavulanate acid (Cft/CA, 0.25/4-0.5/4, 4/4), cefoxitin (Cfx, 8), ceftazidime (Caz, 1-16), ceftazidime-clavulanate acid (Caz/CA, 0.25/4-0.5/4, 2/4), cefuroxime (Crm, 8), ciprofloxacin (Cp, 0.06, 0.25-1), colistin (Cl, 2-4), ertapenem (Etp, 0.12-0.5), fosfomycin (Fos, 8, 32), gentamicin (Gm, 2-4), imipenem (Imp, 2-4), levofloxacin (Lvx, 0.5-1), meropenem (Mer, 0.12, 2-8), nalidixic acid (NA, 16), nitrofurantoin (Fd, 64), norfloxacin (Nxn, 0.5-1), piperacillin-tazobactam (P/T, 8/4-16/4), tobramycin (To, 2-4), and trimethoprim-sulfamethoxazole (T/S, 2/38-4/76). Isolates were categorized as susceptible, intermediate, or resistant to each tested antimicrobial compound following the EUCAST guidelines. Isolates 1-DH1, 3-DH2, and 4-DH3 were selected for WGS.

### 4.3. Isolates DNA Extraction

For bacterial genomic DNA extraction, isolates 1-DH1, 3-DH2, and 4-DH3 were inoculated into brain heart infusion (BHI) (Liofilchem, Roseto degli Abruzzi, Italy), followed by bead mill homogenization using a TissueLyser (Qiagen, Hilden, Germany) system as previously described [88,89]. Isolate 2-DH1 was not further explored since this presented the same biochemical and AMR profiles as isolate 1-DH1. Briefly, each isolate was inoculated into tubes containing 5 mL of BHI and incubated at 37 °C for 48 h. The tubes were then centrifuged at 4000 rpm for 10 min and 4 mL of the supernatant was discarded. The pellet, along with the remaining BHI, were transferred to new sterile 2 mL tubes, to which 0.1 mm precellys beads (Bertin Technologies SAS, Montigny-le-Bretonneux, France) washed with HCL (1M) were added. Tubes were subsequently placed in a TissueLyser (Qiagen, Hilden, Germany) at 15 Hz for 3 min. Genomic DNA was then extracted using the Mag-Bind^®^ Universal Pathogen Core Kit-Tissue protocol (OMEGA BIO-TEK, Norcross, GA, USA) and quantified using the 1x ds DNA HS Assay Kit (TransGen Biothec, Beijing, China) in a Qubit™ fluorometer (Invitrogen, Carlsbad, CA, USA), following the manufactures instructions.

### 4.4. Whole-Genome Sequencing

Genomic DNA from isolates 1-DH1 and 3-DH2 (both with DNA concentration > 60.00 ng/μL) and isolate 4-DH3 (with DNA concentration equal to 48.30 ng/μL) were sequenced by Plasmidsaurus (Bacterial Genome sequencing, London, UK) using Oxford Nanopore Technology (ONT, Oxford, UK) via a PromethION 24 instrument, equipped with an R10.4.1 flow cell. The library preparation utilized the Native Barcoding Kit 96 V14 (SQK-NBD114.96, Oxford Nanopore Technologies, Oxford, UK), following the manufacturer’s instructions [90], followed by custom analysis and annotation. The raw FASTQ reads were basecalled in super-accurate mode, using ont-doradod-for-promethion v7.4.12, applying a Q-score of minimum 10, with adapters and barcodes trimmed via MinKNOW v6.0.4. Initial quality control of the sequencing reads was performed using NanoPlot 1.43.0 [91]. Sequencing adapters and barcodes were removed using Porechop 0.2.4 [92]. To filter the reads, NanoFilt v.2.8.0 [91] was employed, applying a minimum average quality score threshold of 10.

### 4.5. Whole-Genome Analysis

Long-read sequencing data were first quality-filtered by removing the bottom 5.00% of reads using Filtlong v0.2.1 [93] with default parameters. The reads were then downsampled to 250 Mb with Filtlong [29] to generate a rough assembly using Miniasm v0.3 [94]. Based on the resulting Miniasm assembly, reads were re-downsampled to approximately 100× genome coverage (if sufficient coverage was available), prioritizing the removal of low-quality reads using Filtlong with a high mean quality weight (—mean_q_weight 10). The filtered reads were assembled using Flye v2.9.1 [95], with parameters optimized for high-quality ONT reads. Assembly polishing was performed with Medaka v1.8.0 [96] using the high-quality reads from the previous step.

The resulting assembly underwent functional and structural evaluation, including genome annotation with Bakta v1.6.1 [97], and assessment of genome completeness and contamination with CheckM v1.2.2 [98]. Taxonomic and plasmid identification were performed using Mash v2.3 [99] against the RefSeq genome and plasmid database [100]. Additionally, plasmid-related sequences were analyzed using PlasmidFinder 2.1 [43], and the MOBscan [101] server. Antimicrobial resistance and VF related genes were screened using ABRicate v1.0.0 with the ResFinder [102], National Center for Biotechnology Information (NCBI) [100], and Comprehensive Antibiotic Resistance Database (CARD) [103] databases for AMR genes, and the Virulence Factor Database (VFDB) [104] for VF-associated genes. The three draft genome sequences of *Serratia marcescens* isolates were deposited in GenBank under the BioProject accession PRJNA1258987. For isolate 1-DH1, the accessions are SUB15321111, SAMN48480600, contig_1, and CP193986; for isolate 3-DH2, the accessions are SUB15321111, SAMN48480601, contig_1_1, and CP193987; and, for isolate 4-DH3, the accessions are SUB15321111, SAMN48480602, contig_2, and CP193988. Species from the NCBI RefSeq database were restricted to entries published within the last two years (as of 24 June 2025). In addition, two whole-genome sequence references from *S. marcescens* isolates recovered from BM cases were included to assess the phylogenomic positioning of host-specific strains. Genome annotation was performed using Bakta (v1.11.1) [97]. All annotated genomes were processed with Panaroo (v1.5.2) [105] using strict filtering parameters to construct a high-confidence pangenome. The built-in core genome alignment was extracted directly from Panaroo and used as the basis for phylogenomic inference. Phylogenomic reconstruction was conducted using IQ-TREE2 (v2.4.0) [106]. The best-fit nucleotide substitution model was determined using the Bayesian Information Criterion (BIC), which selected GTR + F + I + R8. To assess the robustness of the tree topology, 1000 ultrafast bootstrap replicates were performed. The resulting tree was visualized using the Interactive Tree of Life (iTOL) (v7) [107]. Isolation source was mapped onto the tree to aid in the interpretation of clustering patterns.

### 4.6. Statistical Analysis

Descriptive statistics were performed using Microsoft^®^ Excel^®^ 2016. Categorical variables were summarized as percentages.

## 5. Conclusions

In conclusion, the Neg-Urine-Combo98 panel used with the MicroScan WalkAway Plus system may serve as a useful tool for the phenotypic identification and antimicrobial susceptibility testing (AST) of Gram-negative isolates from clinical mastitis (CM) and subclinical mastitis (SCM) bovine mastitis (BM) cases. In this study, while the AST results were reliable, the phenotypic identification of Serratia species was less consistent. Notably, isolate 3-DH2 was misidentified phenotypically as *Serratia odorifera*. Whole-genome sequencing (WGS) provided higher specificity, correctly identifying all tested isolates (including 3-DH2) as *S. marcescens*, thus addressing the limitations of phenotypic methods. Phenotypic AST revealed resistance to tobramycin (To), clindamycin (Crm), chloramphenicol (Cl), and fidaxomicin (Fd) in *S. marcescens* isolates from BM, representing a novel resistance profile not previously reported. Conversely, susceptibility to antimicrobials such as cefepime (Cpe), cefotaxime (Cft), ceftazidime/clavulanic acid (Caz/CA), ertapenem (Etp), imipenem (Imp), meropenem (Mer), nalidixic acid (NA), ciprofloxacin (Cp), levofloxacin (Lvx), norfloxacin (Nxn), amikacin (AK), gentamicin (Gm), trimethoprim/sulfamethoxazole (T/S), azithromycin (AZ), cefoxitin (Cfx), ceftazidime (Caz), fosfomycin (Fos), cefotaxime/clavulanic acid (Cft/CA), and piperacillin/tazobactam (P/T) suggests potential therapeutic options.

A whole-genome sequencing analysis of *S. marcescens* isolates 1-DH1, 3-DH2, and 4-DH3 revealed the presence of AMR genes including *AAC(6′)-Ic*, *aac(6′)-Ic_1*, *aac(6′)-Ial*, *H-NS*, *SRT-2*, *oqxB*, *oqxB_1*, *oqxB25*, *mexI*, *CRP*, and *blaSST-1*, as well as virulence factor (VF) genes *flgH*, *fliP*, *fliM*, and *fliG*. To the best of our knowledge, this is the first report of these AMR and VF genes in *S. marcescens* isolates from bovine mastitis cases. Furthermore, a phylogenetic analysis indicated that the genomes of these isolates are more closely related to *S. marcescens* strains originating from environmental sources than to those from human or insect cases. Overall, these findings raise concerns relevant to public health, One Health, and zoonotic disease fields.

In future studies, investigations involving a larger number of *S. marcescens* isolates from each studied DH are needed to better understand local antimicrobial resistance patterns. Additionally, environmental sampling, including bedding, water, milking equipment, and potential human and animal reservoirs, should be conducted in order to identify possible sources of contamination and transmission within the frameworks of public health, One Health, and zoonotic disease risk. Furthermore, future research could explore the efficacy of natural disinfectants with antibiofilm properties as potential control strategies.

## Figures and Tables

**Table 1:** Phenotypic identification and antimicrobial susceptibility testing obtained from the four isolates from raw milk cows with BM by MicroScan WalkAway Plus.

Neg-Urine-Combo98 Panel	Isolate 1-DH1	Isolate 2-DH1	Isolate 3-DH2	Isolate 4-DH3
**Bacteria Identification** **% of probability**	*S. marcescens* 99.99	*S. marcescens* 99.99	*S. odorifera* 94.66	*S. marcescens* 99.99
**Antimicrobials**				
**β-lactam**				
Amoxicillin-clavulanate acid-E (Aug-E)	R * (>32.00)	R * (>32.00)	R * (>32.00)	R * (32.00)
Ampicillin (AM)	R * (>8.00)	R * (>8.00)	R * (>8.00)	R * (>8.00)
Cefepime (Cpe)	S * (≤1.00)	S * (≤1.00)	S * (≤1.00)	S * (≤1.00)
Cefotaxime (Cft)	S * (<1.00)	S * (<1.00)	S * (<1.00)	S * (<1.00)
Ceftazidime-clavulanate acid (Caz/CA)	S * (≤0.25)	S * (≤0.25)	S * (≤0.25)	S * (≤0.25)
Ertapenem (Etp)	S * (≤0.12)	S * (≤0.12)	S * (≤0.12)	S * (≤0.12)
Imipenem (Imp)	S * (≤2.00)	S * (≤2.00)	S * (≤2.00)	S * (≤2.00)
Meropenem (Mer)	S * (≤0.12)	S * (≤0.12)	S * (≤0.12)	S * (≤0.12)
**Quinolone**				
Nalidixic Acid (NA)	S * (≤0.16)	S * (≤0.16)	S * (≤0.16)	S * (≤0.16)
Ciprofloxacin (Cp)	S * (0.25)	S * (0.25)	S * (0.25)	S * (≤0.06)
Levofloxacin (Lvx)	S * (≤0.50)	S * (≤0.50)	S * (≤0.50)	S * (≤0.50)
Norfloxacine (Nxn)	S * (≤0.50)	S * (≤0.50)	S * (≤0.50)	S * (≤0.50)
**Aminoglycoside**				
Amikacin (AK)	S * (≤8.00)	S * (≤8.00)	S * (≤8.00)	S * (≤8.00)
Gentamicin (Gm)	S * (≤0.20)	S * (≤0.20)	S * (≤0.20)	S * (≤0.20)
Tobramycin (To)	R * (4.00)	R * (4.00)	R * (4.00)	R * (≤0.20)
**Sulphonamide**				
Trimethoprim-Sulfamethoxazole (T/S)	S * (≤2/38)	S * (≤2/38)	S * (≤2/38)	S * (≤2/38)
**Monobactam**				
Aztreonam (AZT)	S * (≤1.00)	S * (≤1.00)	S * (≤1.00)	S * (≤1.00)
**Cephalosporin**				
Cefoxitin (Cfx)	R * (≤8.00)	R * (≤8.00)	S * (≤8.00)	R * (≤8.00)
Ceftazidime (Caz)	S * (≤1.00)	S * (≤1.00)	S * (≤1.00)	S * (≤1.00)
Cefuroxime (Crm)	R * (>8.00)	R * (>8.00)	R * (>8.00)	R * (>8.00)
**Polymyxin**				
Colistin (Cl)	R * (>4.00)	R * (>4.00)	R * (>4.00)	R * (>4.00)
**Phosphonic**				
Fosfomycin (Fos)	S * (≤8.00)	S * (≤8.00)	S * (≤8.00)	S * (≤8.00)
**Nitrofuran**				
Nitrofurantoin (Fd)	R * (>64.00)	R * (>64.00)	R * (>64.00)	R * (>64.00)
**Cephalosporin/β-lactam**				
Cefotaxime-clavulanate acid (Cft/CA)	S * (≤0.25)	S * (≤0.25)	S * (≤0.25)	S * (≤0.25)
**Penicillin/β-lactam**				
Piperacillin-Tazobactam (P/T)	S * (≤8.00)	S * (≤8.00)	S * (≤8.00)	S * (≤8.00)
**Total**	25	25	25	25
**% Susceptible**	72.00	72.00	76.00	72.00
**% Intermediate**	0.00	0.00	0.00	0.00
**% Resistant**	28.00	28.00	24.00	28.00

DH (Dairy herd), S (Susceptible), R (Resistant), * (Minimum inhibitory concentrations (MICs) achieved in this study).

**Table 2:** Genome characterization obtained for the three isolates, 1-DH1, 3-DH2, and 4-DH3, by WGS.

Isolate	Length (bp)	Bacterial Species	% Identity
1-DH1	5,083,761	NZ_JGVB01000001.1 *Serratia marcescens* strain ATCC 14041 contig001	99.30
3-DH2	5,083,758	NZ_JGVB01000001.1 *Serratia marcescens* strain ATCC 14041 contig001	99.30
4-DH3	5,082,903	NZ_JGVB01000001.1 *Serratia marcescens* strain ATCC 14041 contig001	99.30

DH (Dairy herd), bp (Base pair).

**Table 3:** Antimicrobial resistance and VF genes identified in the *S. marcescens* whole-genome sequences of the three isolates, 1-DH1, 3-DH2, and 4-DH3, by ABRicate (BLAST) using CARD, ResFinder and NCBI, and VFDB databases, respectively.

Database		CARD			NCBI			ResFinder		* AMR in CARD, NCBI or ResFinder
AMR Gene/Isolate	1	3	4	1	3	4	1	3	4	
*AAC(6′)-Ic*	x	x	x							Aminoglycoside
*aac(6′)-Ic_1*							x	x	x	Amikacin; tobramycin
*aac(6′)-Ial*				x	x	x				Aminoglycoside
*H-NS*	x	x	x							Cephalosporin, fluoroquinolone, macrolide, penicillin, tetracycline, cephamycin
*tet(41)*	x	x	x	x	x	x	x	x	x	Acycline, doxycycline, tetracycline
*SRT-2*	x	x	x							Cephalosporin, cefotaxime
*oqxB*	x	x	x							Diaminopyrimidine, fluoroquinolone, glycylcycline, nitrofuran, tetracycline
*oqxB_1*							x	x	x	Nalidixic acid; ciprofloxacin
*oqxB25*				x	x	x				Phenicol, quinolone
*mexI*	x	x	x							Acridine_dye, fluoroquinolone, tetracycline
*CRP*	x	x	x							fluoroquinolone, macrolide, penicillin
*blaSST-1*				x	x	x				Cephalosporin
*blaSST-1_1*							x	x	x	N/A
**Database**					**VFDB**					** VF in VFDB
**VF gene/Isolate**		**1**			**3**			**4**		
*fliG*		x			x			x		Flagellar motor switch protein G
*fliM*		x			x			x		Flagellar motor switch protein FliM
*fliP*		x			x			x		Flagellar biosynthetic protein FliP
*flgH*		x			x			x		Flagellar L-ring protein precursor FlgH

AMR (Antimicrobial resistance), VF (Virulence factor), x (Gene presented), N/A (Not available), CARD (Comprehensive Antibiotic Resistance Database), NCBI (National Center for Biotechnology Information), * AMR genes results obtained in CARD, NCBI, or ResFinder could be seen in Table S2, ** VF genes results obtained in VFDB (Virulence Factor Database) could be seen in Table S2.

## Data Availability

The original contributions presented in this study are included in the article. Further inquiries can be directed to the corresponding author.

## Supplementary Materials

The following supporting information can be downloaded at: https://www.mdpi.com/article/10.3390/antibiotics14090892/s1. Table S1. Clusters of orthologous groups (COG) obtained in the genome analysis of the three isolates by WGS. Table S2. Results obtained for sequences placed in the CARD, NCBI and ResFinder, and VFDB databases for AMR genes and VF identification, respectively, from WGS of isolates 1-DH1, 3-DH2 and 4-DH3.
