# Supplementary material for: Serratia marcescens Isolates from Bovine Mastitic Milk: Antimicrobial Resistance and Virulence Features
Source: Antibiotics (Basel). 2025 Sep 3;14(9):892. doi: 10.3390/antibiotics14090892 (PMC12466401; doi:10.3390/antibiotics14090892)
Supplement: Supplementary file 1 [file antibiotics-14-00892-s001.zip › Table S1.pdf]

Table S1. Clusters of orthologous groups (COG) obtained in the genome analysis of the three isolates by WGS.

Character	COG
**D**	Cell cycle control, division, chromosome partitioning
**M**	Cell wall/membrane/envelope biogenesis
**N**	Cell motility
**O**	Post-translational modification, protein turnover, chaperones
**T**	Signal transduction mechanism
**U**	Intracellular trafficking, secretion, and vesicular transport
**V**	Defense mechanism
**W**	Extracellular structures
**Y**	Nuclear structure
**Z**	Cytoskeleton
**A**	RNA processing and modification
**B**	Chromatin structure and dynamics
**J**	Translation, ribosomal structure, and biogenesis
**K**	Transcription
**L**	Replication, recombination, and repair
**X**	Mobilome: prophages, transposons
**C**	Energy production and conversion
**E**	Amino acid transport and metabolism
**F**	Nucleotide transport and metabolism
**G**	Carbohydrate transport and metabolism
**H**	Coenzyme transport and metabolism
**I**	Lipid transport and metabolism
**P**	Inorganic ion transport and metabolism
**Q**	Secondary metabolites biosynthesis, transport, and metabolism
**R**	General function prediction only
**S**	Function unknown
